# Development and Characterization of Sodium Bicarbonate-Based Gel for Cytolytic Vaginosis

**DOI:** 10.3390/pharmaceutics16111436

**Published:** 2024-11-11

**Authors:** Carlos Gaspar, Ana Sofia Agonia, Sara Felício, Mariana Tomás, Diana Esteves, Rita Palmeira-de-Oliveira, Gilbert G. G. Donders, José Martinez-de-Oliveira, Ana Palmeira-de-Oliveira

**Affiliations:** 1 CICS-UBI—Health Sciences Research Center, University of Beira Interior, Av. Infante D. Henrique, 6200-358 Covilhã, Portugal; cgaspar@fcsaude.ubi.pt (C.G.); sofiagonia@gmail.com (A.S.A.); mariana.tomas@ubi.pt (M.T.); rpo@fcsaude.ubi.pt (R.P.-d.-O.); jmo@fcsaude.ubi.pt (J.M.-d.-O.); 2 Faculty of Health Sciences, University of Beira Interior, Av. Infante D. Henrique, 6200-358 Covilhã, Portugal; 3 Labfit—HPRD Health Products Research and Development, Lda, Edifício UBIMEDICAL Estrada Municipal 506, 6200-284 Covilhã, Portugal; sara.felicio@labfit.eu (S.F.); diana.esteves@labfit.eu (D.E.); 4 Femicare Clinical Research for Women, 3300 Tienen, Belgium; gilbert.donders@gmail.com; 5 Department of Obstetrics and Gynecology, University of Antwerp, 2550 Edegem, Belgium; 6 Department of Obstetrics and Gynecology, Regional Hospital, 3300 Tienen, Belgium

**Keywords:** cytolytic vaginosis, *Lactobacillus*, sodium bicarbonate, vagina, gels

## Abstract

**Background/Objectives:** Cytolytic vaginosis or, classically, Doderlein’s cytolysis is characterized by significant growth of species of the *Lactobacillus* genus, which leads to high amounts of lactic acid in the vaginal environment. *Lactobacillus crispatus* has been proposed as a key pathogen in this clinical condition. The symptomatology of cytolytic vaginosis is commonly confused with that of vulvovaginal candidosis, leading to inadequate and ineffective azole therapies. Nevertheless, historically, the use of sodium bicarbonate intimate baths was an effective way to reduce the symptoms of cytolytic vaginosis. **Methods:** In this study, four HPMC gel prototypes were developed, containing sodium bicarbonate concentrations ranging from 4% to 7% (*w/w*). These gels were evaluated for their physicochemical properties, antimicrobial activity, interference with lactobacilli adhering to cells, and cellular and tissue biocompatibility. **Results:** The gels presented pH values of around 9.0, and osmolality between 706 mOsm/kg (F4) and 1065 mOsm/kg (F7). The viscosity upon heating to physiologic temperature and dilution with simulated vaginal fluid was highly affected by the concentration of sodium bicarbonate. Gels with higher sodium bicarbonate concentrations (F6 and F7) were not shown to be stable in these conditions. All formulations exhibited effective antimicrobial activity against seven *L. crispatus* strains, with MIC values ranging from 6.25% to 25% (*v/v*) in terms of dilution. Additionally, the 4% (*w/w*) gel significantly interfered with the adhesion of *L. crispatus* to epithelial cells in competition and exclusion assays, reducing adhesion by more than 90% in relation to the control. Cytotoxicity tests on the Hec-1A, HeLa, and VK2/E6E7 cell lines indicated that the F4 and F5 gels demonstrated lower cytotoxicity levels compared to those with higher concentrations. Furthermore, ex vivo assays using porcine vaginal tissue confirmed that the 4% gel was non-toxic at a 25% (*v/v*) dilution. **Conclusions**: Based on these results, the 4% (*w/w*) sodium bicarbonate gel (F4) emerges as a promising therapeutic option for cytolytic vaginosis, offering effective bacterial interference, favourable physicochemical properties, and biocompatibility suitable for vaginal application.

## 1. Introduction

Cytolytic vaginosis is a clinical condition characterized by the presence of a high number of lactobacilli, together with lysed epithelial cells and free nuclei, in the vaginal environment, as well as an extremely low vaginal fluid pH [1,2]. The *Lactobacillus* genus is characterized by pleomorphic, Gram-positive, aerobic or facultative anaerobic, and non-spore-forming bacteria [3]. About 250 species belonging to this genus have been described so far, and more recently, some have been reclassified into new different genera [4]. 

Recent studies suggest that *Lactobacillus crispatus*, either alone or in combination with other bacteria, may be responsible for cytolytic vaginosis [5,6]. This hypothesis contrasts with the concept that vaginal health is related to the prevalence of lactic acid bacteria [7,8,9]. Thus, in the case of cytolytic vaginosis, this may not necessarily indicate a cause-effect relationship. In fact, until now, little has been known about the pathogenesis of cytolytic vaginosis. Assuming that *Lactobacillus* are the pathogens, it is not clear why this infection is not more prevalent, these being the bacteria most prevalent in the vaginal ecosystem. The answer may lie in the microorganism–host interaction, including the understanding of whether this is a species- or strain-related condition of endogenous or external origin. Furthermore, some studies suggest an association between cytolytic vaginosis and cervical abnormalities, reporting the prevalence of cytolytic vaginosis in 12% of women with carcinoma in situ, 9% in women with dysplasia, and 4% in women with invasive carcinoma, compared to 19% in women without cervical abnormalities [10]. A recent comprehensive review raised relevant points about the prevalence of cytolytic vaginosis in women with cervical dysplasia, as well as its implications for cervical health [11].

The clinical symptomatology of cytolytic vaginosis is characterized by pruritus, dyspareunia, and vulvar dysuria, with the exacerbation of these symptoms during the luteal phase [1,12]. The diagnosis of this clinical condition considers the grade of suspicion; the absence of the known etiologic agents responsible for infections, such as *Trichomonas vaginalis*, *Gardnerella vaginalis*, and *Candida* spp., on wet smear; a high number of *Lactobacillus* spp.; a low number of leukocytes; evidence of the dissolution of the cytoplasm of the intermediate vaginal epithelium; and the pH of the vaginal exudate being between 3.5 and 4.5 [1]. The symptoms and appearance of the vaginal exudate are similar to those of vulvovaginal candidosis, leading physicians to misdiagnose and start azoles-based treatment, which is ineffective at addressing cytolytic vaginosis. So far, there is no therapeutic commercial solution available on the market that has been specifically developed for the treatment of cytolytic vaginosis. In fact, in clinically suspicious cases, gynecologists empirically prescribe genital baths or irrigations with sodium bicarbonate solutions (30 to 60 g of sodium bicarbonate in approximately 1 L of water) two to three times per week and then once or twice a week [1,13,14]. The aim is to promote an increase in the local pH to control the symptoms that are related to the low pH present in this clinical condition. However, these treatments are not standardized. Additionally, vaginal irrigations are also not considered appropriate approaches as they promote the disruption of the vaginal microbiome.

Vaginal gels with sodium bicarbonate have been proposed by our research group as a treatment for vulvovaginal candidosis due to the inherent antifungal properties of sodium bicarbonate [15]. In that study, gels based on carbomer and hydroxypropyl methylcellulose were tested regarding their antifungal activity against *Candida* spp. The formulation that showed the highest potential for application contained 5% (*w/w*) sodium bicarbonate and 1% (*w/w*) carbomer (Formulation C), demonstrating antifungal activity with an MIC of 2.5% (*v/v*) against *C. albicans*, *C. glabrata*, *C. krusei*, *C. tropicalis*, and *C. parapsilosis* [15]. While being widely available for other therapeutic purposes, including the treatment of systemic and gastrointestinal clinical conditions (such as metabolic acidosis, diabetic ketoacidosis, pyrosis and dyspepsia, electrolyte imbalances, and acute kidney injury) [16,17,18,19,20], topical formulations of sodium bicarbonate for vaginal conditions are not yet available on the market. 

The rational design of vaginal products, integrating efficacy, safety, quality, and compliance (preferences) attributes, is essential for therapeutic success. It is known that women consider the vaginal route as safe and effective in terms of treating local conditions, but still complain of several limitations related to comfort that influence their use of vaginal products [21,22].

In this work, we further explore the potential of hydroxypropyl methylcellulose-based vaginal formulations to design a new therapeutic product for the local treatment of cytolytic vaginosis. Furthermore, we studied the mechanistic effect of sodium bicarbonate gels as therapeutic agents for treating cytolytic vaginosis in view of recent scientific insights on the role of *L. crispatus.* We also assessed the safety of such formulations for vaginal applications through in vitro and ex vivo models.

## 2. Materials and Methods

### 2.1. Gels

Gels were developed as simple formulations based on hydroxypropyl methylcellulose dispersion (HPMC; Methocel K100M—viscosity 75,000–140,000 cP, 2% aqueous solution; 20 °C; Dow, Midland, MI, USA) and they differed in terms of the concentration of sodium bicarbonate. For gel preparation, sodium bicarbonate (purity level > 99.7%) (Sigma-Aldrich, Darmstadt, Germany) solutions were prepared with ultrapure water (obtained in house using Merck Milli-Q^®^ reference equipment) at the final defined concentration and then the polymer was gradually dispersed using a helical stirrer (Heidolph RZR 2041, Heidolph Instruments GmbH & Co., Schwabach, Germany) at 500 rpm. 

Table 1 shows the qualitative and quantitative compositions of these gels, which were numbered (F4–F7) based on the *w/w* concentration of sodium bicarbonate.

### 2.2. Characterization of the Gels

#### 2.2.1. pH

pH determination was performed directly on the gels and after their dilution with simulated vaginal fluid with an acidic pH (3.5), which was chosen to comply with the pathological features of cytolytic vaginosis. For that purpose, 3 g of the product (considered as the application dose to be defined) was diluted in 0.825 mL of simulated vaginal fluid with mucin (mVFS). This procedure was designed to simulate the pH of these gels after contact with vaginal fluid, since 0.75 mL is the estimated mean volume of vaginal fluid at any moment. We assumed an increase of 10% in that volume, in accordance with the previous studies of our group with bicarbonate gels for vulvovaginal candidosis, since these conditions are often confused and show similar chemical profiles [15,23]. Measurements were performed, in triplicate, using a pH meter (Seven Compact S220 pH/Ion meter, Mettler Toledo, Columbus, OH, USA), at room temperature (25 °C ± 0.2 °C) and at vaginal physiologic temperature (37 °C ± 0.2 °C).

mVFS was prepared according to the recipe of Owen and Katz [24] added mucin 1.5% (*w/v*) to include the cervical component that contributes to the bioadhesive profiles of products [15,23,25]. So, 3.51 g of sodium chloride (NaCl, JT Baker, Phillipsburg, NJ, USA); 1.4 g of potassium hydroxide (KOH, VWR, Fontenay-sous-Bois, France); 0.22 g of calcium hydroxide [Ca(OH)_2_), Acros Organics, Fair Lawn, NJ, USA]; 0.018 g of Bovine Serum Albumin (BSA, Sigma-Aldrich, Darmstadt, Germany); 2.00 g of lactic acid (Sigma-Aldrich, Darmstadt, Germany); 1.00 g of acetic acid (Thermo Fischer Scientific, Waltham, MA, USA); 0.16 g of glycerol (Acofarma, Terrassa, Spain); 0.4 g of urea (VWR, Fontenay-sous-Bois, France); 5.00 g of glucose (VWR, Fontenay-sous-Bois, France); and 15.00 g of porcine gastric mucin type II (Sigma-Aldrich, Darmstadt, Germany) were added to slightly less than 1 L of ultrapure water and stirred mechanically until complete dissolution [23]. The pH of the mixture was then adjusted to 4.2 using sodium hydroxide, and the final volume was adjusted to 1 L. Immediately before the experiments, the pH was readjusted to 3.5 with lactic acid. 

#### 2.2.2. Osmolality

Osmolality was determined in triplicate using a freezing point osmometer (Micro-Osmometer, Model 3300, Advanced Instruments, Norwood, MA, USA), as previously described [26,27], on a 20 μL aliquot. Standardization was performed using three standards: distilled water (zero point), 50 mOsm/Kg, and 850 mOsm/Kg. These are commercially available from the equipment manufacturer. Undiluted samples were tested directly for osmolality. Further dilutions with deionized water (1:2) were performed to allow for measurements whenever the osmolality values were above the maximum limit of the calibration curve. In these cases, the osmolality values were multiplied by the dilution factor. Furthermore, osmolality was determined after the dilution of 3 g of product with 0.825 mL of mVFS using the same approach described for pH measurements (see Section 2.2.1). If needed, further dilutions were performed with distilled water, as described for undiluted samples.

#### 2.2.3. Viscosity

Viscosity was assessed using a cone–plate rheometer (Brookfield DV-2 T, Brookfield, Middleboro, MA, USA), using 0.5 mL of the formulation [15,28]. Viscosity measurements were performed at room temperature (25 ± 0.2 °C) and at vaginal physiologic temperature (37 ± 0.2 °C). Formulations F4 and F5 were further tested for viscosity changes upon dilution with mVFS (3 g of gel with 0.825 mL mVFS) at 37 °C.

The cone spindle used in the rheometer was the CPA-52Z (Brookfield, Middleboro, MA, USA), with a cone angle and radius of 3° and 1.2 cm, respectively.

The viscosity was determined using shear rates of 300 s^−1^ (torque 10–100%). The elapsed time of each determination was set so that the cone completed at least 4 rotations in each reading. Tests were performed in triplicate. 

Formulations were left to rest for 1 min between measurements [29].

#### 2.2.4. Preliminary Stability Studies

Preliminary stability testing was performed to assess gel behaviour when subjected to stress conditions. So, temperature cycling tests were performed for gel F7 (the worst-case scenario regarding bicarbonate concentration) by subjecting the gel to 24 h storage at alternating extreme temperatures. Gel in glass packaging was stored at 40 ± 2 °C (Binder incubator, Binder GmbH, Tuttlingen, Germany) for 24 h, and then these conditions were changed to 4 ± 2 °C for 24 h, for a total period of 4 weeks. This method is used to stress formulations and assess changes that may occur in these conditions [30,31]. After 4 weeks, the sensorial (organoleptic) characteristics of the formulations were assessed and compared with the ones obtained at timepoint 0. A glass flask of each formulation was maintained at room temperature and at 5 °C for 1 month to study their behaviours at these temperatures. 

### 2.3. Antimicrobial Susceptibility Testing

#### 2.3.1. *Lactobacillus crispatus* and Growth Conditions

The bacterial strains used in both the antimicrobial tests and tests of adhesion to epithelial cells were isolated from the vaginas of healthy women (n = 3) and women diagnosed with cytolytic vaginosis (n = 4) (Table 2). No patient recruitment or selection was conducted for this study. Instead, convenience samples were used, collected exclusively for diagnostic purposes during or after vulvovaginal clinical episodes. This study was approved by the Ethics Committee of the University of Beira Interior (CE-UBI-Pj-2018-024). Bacterial isolates were previously identified by multiplex PCR specific to species identification [32] and subsequently confirmed by 16S rRNA gene sequencing.

The strains were kept at −80 °C (Infrico Medcare, Córdoba, Spain) and recovered in de Man Rogosa Sharpe (MRS) broth (VWR Prolabo, VWR, Fontenay-sous-Bois, France) at 37 °C for 48 h in anaerobic conditions. Prior to use, they were sub-cultured on MRS agar (VWR Prolabo, VWR Fontenay-sous-Bois, France) and incubated at 37 °C for 24 to 48 h in anaerobic conditions. All incubations were performed in an incubator (Binder GmbH, Tuttlingen, Germany) with a controlled atmosphere of CO_2_ (10%) and O_2_ (1%). 

#### 2.3.2. Determination of Minimal Inhibitory Concentration (MIC)

The antimicrobial effect of the samples described above (Section 2.1) and of the sodium bicarbonate solution against *L. crispatus* (described in Table 2) was tested using the broth microdilution method described by the European Committee for Antimicrobial Susceptibility Testing (EUCAST) of the European Society of Clinical Microbiology and Infectious Diseases (ESCMID) [33]. Briefly, the test samples were diluted in a 96-well flat-bottom microplate (VWR Collection, VWR, Fontenay-sous-Bois, France) following two-fold serial dilution with MRS broth medium. The final concentration of the sodium bicarbonate solution tested ranged from 0.78 to 50.00 mg/mL and dilutions of the gel base and gels ranged from 0.78 to 50.00% (*v/v*). Before testing, strains were inoculated in MRS agar medium and incubated for about 18 h at 37 °C in anaerobic conditions. Bacterial suspensions were adjusted to 0.5 McFarland (Grant Instruments, Shepreth, UK) and then diluted in the culture medium to obtain a final cell concentration of 5 × 10^5^. The 96-well plates containing 100 µL of the test samples were inoculated with 100 µL of the bacterial suspension. Microplate wells with and without test samples were used as negative and positive controls of microbial growth, respectively. The plates were incubated for 24 h at 37 °C in anaerobic conditions, and the visual MIC was assessed as the lowest concentration of the test sample that visibly inhibited microbial growth. Additionally, optical densities measured (Promega, Madison, WI, USA) at 600 nm were used to determine the MIC_50_ and MIC_90_, using positive control as reference. 

All assays were performed in triplicate in three independent tests.

#### 2.3.3. Determination of Minimal Bactericidal Concentration (MBC)

To determine MBC, 5 μL of the content of wells without visible bacterial growth were inoculated in MRS agar medium plates. The plates were incubated for 48 h at 37 °C in anaerobic conditions (Binder GmbH, Tuttlingen, Germany) and inspected for the presence of bacterial growth (colony-forming units). The minimal concentration of the testing samples that exhibited the absence of growth was considered the MBC.

#### 2.3.4. Effect of pH on *Lactobacillus crispatus* Growth

In order to elucidate the mechanism of action of sodium bicarbonate on *L. crispatus* strains, the strains were placed in contact with MRS culture medium with the pH adjusted to 8.6. This pH value was previously determined by measuring the 50% (*v/v*) dilution of the bicarbonate sodium gel in MRS culture medium. The presence or absence of microbial growth was evaluated after 24 h of incubation at 37 °C in anaerobic conditions. Additionally, the optical density was measured at 600 nm and compared to the positive control.

### 2.4. In Vitro Cytotoxicity Testing

#### 2.4.1. Cell Lines and Culture Conditions

The human cell lines HEC-1A (HTB-112™), HeLa (CCL-2™), and VK2 E6/E7 (CRL-2616™), acquired from American Type Culture Collection (ATCC), were used to test the in vitro cytotoxicity of the testing samples. These epithelial cell lines represent distinct anatomical regions of the female reproductive tract: the uterus, cervix, and vagina. The uterine cell line Hec-1A, isolated from human endometrial adenocarcinoma, was grown in McCoy’s 5A medium (Sigma-Aldrich, Darmstadt, Germany) supplemented with 100 U/mL penicillin, 100 mg/mL streptomycin (Gibco, Thermo Fisher Scientific, Waltham, MA, USA), and 10% fetal bovine serum (FBS) (Gibco, Thermo Fisher Scientific, Waltham, MA, USA). The cervical HeLa cell line isolated from human cervical adenocarcinoma was grown in DMEM culture medium (Biosera, Nuaille, France) supplemented with 100 U/mL penicillin, 100 mg/mL streptomycin, and 10% FBS. The vaginal VK2 E6/E7 cell line isolated from endometriosis was cultured in serum-free keratinocyte culture medium (Gibco, Thermo Fisher Scientific, Waltham, MA, USA) with 0.1 ng/mL human recombinant epithelial growth factor (Gibco, Thermo Fisher Scientific, Waltham, MA, USA), 0.05 mg/mL bovine pituitary extract (Gibco, Thermo Fisher Scientific, Waltham, MA, USA), and calcium chloride 44.1 mg/L (Labchem, Pittsburgh, PA, USA). The cells were maintained at 37 °C with 5% CO_2_ in a humid atmosphere (Binder GmbH, Tuttlingen, Germany). Sub-cultivation was performed according to the supplier’s recommendations. All morphological changes and the growth profile were monitored by observation under the inverted microscope VisiScope^®^ IT415 PH (VWR Collection, VWR, Fontenay-sous-Bois, France). 

#### 2.4.2. Cellular Cytotoxicity—MTT Method

The MTT reduction method was performed according to ISO 10993-5 [34]. Briefly, 10^4^ cells per well were seeded in 96-well flat-bottomed microplates (VWR Collection, VWR, Fontenay-sous-Bois, France) using the respective complete culture medium of each cell line. The cells were allowed to adhere for 24 h at 37 °C with 5% CO_2_ (Binder GmbH, Tuttlingen, Germany) in a humid atmosphere until reaching semi-confluence. The test samples (described in Section 2.1) were diluted in microcentrifuge tubes (VWR Collection, VWR, Fontenay-sous-Bois, France), following two-fold serial dilution with complete culture medium. Then, 100 μL of each dilution were placed in contact with cells. The final tested concentration of the sodium bicarbonate solution ranged from 0.78 to 50.00 mg/mL, and for both the base gel and dilutions of sodium bicarbonate gels ranged from 0.78 to 50 % (*v/v*). The test sample dilutions were left in contact with the cell lines for 24 h at 37 °C with 5% CO_2_ in a humid atmosphere . After this period, the cells were washed with PBS (Thermo Fisher Scientific, Waltham, MA, USA) and incubated for 3 h with an MTT solution at 1 mg/mL (Thermo Fisher Scientific, Waltham, MA, USA). Finally, the formazan crystals were extracted with 100 μL of isopropanol (Acros Organics, Fair Lawn, NJ, USA) for 15 min while protected from the light. The absorbances were measured at 570 nm and 650 nm to remove interferences (background) in a microplate spectrophotometer (BIORAD xMark, Bio-rad, Hercules, CA, USA). The negative control (reference for 100% viability) consisted of complete culture medium without test sample. Then, 2% SDS (Thermo Fisher Scientific, Waltham, MA, USA) was used as a positive control (cytotoxic effect). Additionally, 50% (*v/v*) diluted culture medium (maximum dilution used in the test) was used as the solvent control. For the testing samples, the CC_50_, corresponding to the concentration that induced 50% of cellular death, was calculated. All assays were performed in sextuplicate in three independent tests. 

#### 2.4.3. Effect of pH on the Viability of Cell Lines

In order to elucidate the effect of the sodium bicarbonate on cell viability, HeLa, Hec-1A, and VK2 E6/E7 cell lines were incubated with culture medium with the pH adjusted to 8.3. This pH value was previously determined by measuring the 50% (*v/v*) dilution of sodium bicarbonate gel in the different culture media. The cell lines were left in contact with the adjusted culture medium for 24 h at 37 °C in the presence of 5% CO_2_. Cell viability was evaluated according to the MTT method, as described in Section 2.4.2.

### 2.5. In Vitro Interference of Sodium Bicarbonate in Lactobacillus crispatus Adhesion to Epithelial Cells

The possible interference of the sodium bicarbonate formulation on *L. crispatus* adhesion to epithelial cells was tested using Hec-1A, HeLa, and VK2 E6/E7 cell lines. Tests were performed with *L. crispatus* PB8 as it was the strain with the highest capacity for adhesion (Figure S1) to the three cell lines. Based both on the results obtained from the antimicrobial and the cytotoxicity testing, the sodium bicarbonate 4% (*w/w*) formulation was selected for this assay. To better elucidate the possible interference of the product with the bacteria adhesion, we performed the test using three different approaches, described below in detail: competition, exclusion, and displacement assays.

#### 2.5.1. Competition Assay Between the Formulation and *Lactobacillus crispatus*

This assay evaluated the ability of the sodium bicarbonate 4% (*w/w*) formulation to compete with *L. crispatus* PB8 for the adhesion sites of the epithelial cells. Briefly, 10^4^ cells of each cell line were allowed to adhere. This was performed in a 96-well flat-bottom microplate, for 48 h with 5% CO_2_ in a humid atmosphere, until confluence was reached. Afterwards, the cells were washed three times with culture medium without FBS and antibiotics (basal culture medium) to remove non-adhered cells and antibiotic traces. Then, the cells were treated, for 1 h, with a mixture of 200 μL *L. crispatus* PB8 at 1 × 10^9^ CFU/mL in basal culture medium and the formulation. An experimental control, corresponding to the replacement of the formulation by basal culture medium, was included. After this period, the cells were washed with basal culture medium and lysed with a Triton X-100 0.1% (*v/v*) solution (VWR, Fontenay-sous-Bois, France). Ten-fold serial dilutions were produced in PBS (Thermo Fisher Scientific, Waltham, MA, USA) and inoculated onto MRS agar. The plates were incubated at 37 °C for 48 h in anaerobic conditions, and the number of CFU/mL was determined. Results are expressed as a percentage of adhered bacteria compared to the experimental control. The assays were performed in quadruplicate in two independent tests.

#### 2.5.2. Exclusion Assay of *Lactobacillus crispatus* by the Formulation

This test assessed the ability of the sodium bicarbonate 4% (*w/w*) formulation to block the adhesion sites of the epithelial cells, preventing *L. crispatus* PB8 from adhering. Briefly, 10^4^ cells of each cell line were allowed to adhere in a 96-well flat-bottom microplate for 48 h with 5% CO_2_ in a humid atmosphere until confluence was reached. After this period, the cells were washed three times with culture medium without FBS and antibiotics (basal culture medium) to remove non-adhered cells and antibiotic traces. Then, 100 μL of sodium bicarbonate 4% (*w/w*) formulation was added to the cell line monolayer for 30 min. Afterwards, 100 μL of *L. crispatus* PB8 suspension at 1 × 10^9^ CFU/mL, prepared in basal culture medium, was added and left to incubate for 1 h. An experimental control, corresponding to the replacement of the formulation by basal culture medium, was included. After this period, the cells were washed with basal culture medium and lysed with a Triton X-100 0.1% (*v/v*) solution. Ten-fold serial dilutions were made in PBS and inoculated onto MRS agar. The plates were incubated at 37 °C for 48 h in anaerobic conditions, and the CFU/mL value was determined. The results are expressed as a percentage of adhered bacteria compared to the experimental control. The assays were performed in quadruplicate in two independent tests.

#### 2.5.3. Displacement Assay of *Lactobacillus crispatus* by the Formulation

This assay evaluated the ability of the sodium bicarbonate 4% (*w/w*) formulation to remove/displace the *L. crispatus* PB8 from the epithelial cells. Briefly, 10^4^ cells of each cell line were allowed to adhere, in a 96-well flat-bottom microplate, for 48 h with 5% CO_2_ in a humid atmosphere until confluence was reached. After this period, the cells were washed three times with culture medium without FBS and antibiotics (basal culture medium) to remove non-adhered cells and antibiotic traces. Then, 100 μL of *L. crispatus* PB8 cell suspension at 1 × 10^9^ CFU/mL, prepared in basal culture medium, was added to the cell monolayer and left to adhere for 1 h. Afterwards, 100 μL of sodium bicarbonate 4% (*w/w*) formulation was added and left to incubate for 30 min. An experimental control, corresponding to the replacement of the formulation by basal culture medium, was included. After this period, the cells were washed with basal culture medium and lysed with a Triton X-100 0.1% (*v/v*) solution. Ten-fold serial dilutions were made in PBS and inoculated onto MRS agar. The plates were incubated at 37 °C for 48 h in anaerobic conditions, and the number of CFU/mL was determined. The results are expressed as a percentage of adhered bacteria compared to the experimental control. The assays were performed in quadruplicate in two independent tests.

#### 2.5.4. Microscopic Observation of *Lactobacillus crispatus* Adhesion to Epithelial Cells

The adhesion of the *L. crispatus* strain PB8 to the different cell lines was monitored by light microscopy using Gram staining. Briefly, 10 mm coverslips were placed in 24-well flat-bottom plates. Then, 10^5^ cells of these cell lines were added to each microplate well and allowed to adhere for 48 h in a 5% CO_2_ humid atmosphere. Cell adhesion and confluence were monitored using inverted microscope. Competition, exclusion, and displacement assays were repeated as mentioned above. After the final washing with basal culture medium, the cell lysis process with Triton-100 0.1% was replaced by Gram staining (Merck Millipore, Darmstadt, Germany). The slides were observed and photographed on an inverted microscope at 100× magnification.

### 2.6. Testing in Ex Vivo Vaginal Tissues

Complete porcine genitalia from a six-month-old animal were obtained from a local slaughterhouse. They were transported under refrigeration conditions and handled within three hours since animal’s death. The vaginal tube was isolated from the adjacent organs and the vestibule part (caudal vagina) was removed after a longitudinal vaginal incision [35]. After rinsing the vestibule in DMEM medium solution (Biosera, Nuaille, France), a manual dermatome was used to standardize tissue segment thickness (Watson Skin Graft Knife, BBraun with Aesculap-blades BA718Rm, Bbraun, Melsungen, Germany). The epithelial section was placed on aluminium foil (contact made by the basolateral side) and punched into 3 mm diameter sections using a biopsy punch (Stiefel, GSK, Research Triangle Park, NC, USA). These sections were maintained in DMEM medium solution until use in the tissue toxicity assay. Tissue thickness was measured with a digital micrometer (Vogel, Kevelaer, Germany) to assess uniformity between samples.

For toxicity assay, the fresh explants were subjected not only to negative and positive controls (DMEM medium, Biosera, and SDS 5% (*v/v*) in DMEM medium and VWR, respectively), but also to two-fold serial dilutions of the test formulation in DMEM medium (starting at a dilution of 50% (*v/v*)). The vehicle control (the formulation where sodium bicarbonate was replaced by water) was also introduced in the study design at a 50% (*v/v*) dilution in DMEM medium. The number of explants per sample or control was 8.

On the day of the explants’ preparation, 100 µL of the serial dilutions of the test formulation and controls was applied to the tissue explants that were already placed in a 96-well flat-bottom tissue culture plates. The contact time was about 24 ± 2 h at 37 °C with 5% CO_2_ (APT.line^TM^ C150E2, Binder GmbH, Tuttlingen, Germany), after which the control and test solutions were removed. Then, 100 µL of the 0.5 mg/mL MTT solution (Thermo Fisher Scientific, Waltham, MA, USA), freshly prepared in DMEM medium, was added to each well and incubated for 2 h at 37 °C with 5% CO_2_. The tissues were extracted with 200 µL of isopropanol (Acros Organics, Fair Lawn, NJ, USA) for at least 2 h at room temperature with mild agitation. After MTT extraction, 100 µL of the isopropanol solution was revealed at 560 nm (GloMax^®^ Explorer System, Promega, Madison, WI, USA).

### 2.7. Data Processing and Statistical Analysis

Data were analyzed to produce arithmetic means and standard deviations. Statistical analyses were performed using two-way ANOVA with Bonferroni post-test using GraphPad Prism version 9.3.1 (GraphPad, San Diego, CA, USA). *p*-values lower than 0.05 were considered significant. 

## 3. Results

### 3.1. Characterization of the Gels

The gels were developed as simple formulations based on HPMC that is a cellulose-derivative, generally recognized as safe (GRAS, included in the FDA Inactive Ingredients Database U.S. FDA), and historically used for vaginal application [36]. The base hydrogel was formulated with HPMC, which is believed not to be significantly influenced by pH, is inert, is bioadhesive, and so improves the retention of formulations in the vagina and improves comfort [37,38]. These properties explain its wide use in vaginal formulations. Humectants, such as glycerine or propyleneglycol, were not included in this formulation to avoid their contributions to osmolality, which could impair the overall safety of the gel [39], especially because the content of sodium bicarbonate was expected to make such a high contribution to osmolality based on our previous results [15]. In fact, we had previously explored the potential of a HPMC gel, containing 1% (*w/w*) of polymer and 5% (*w/w*) of sodium bicarbonate, that was to be used for VVC treatment. In that study, the said formulation (D) was shown to have low viscosity but an interesting bioadhesive profile. For this reason, we decided to further explore this polymer as the viscosity agent of these gels, increasing its concentration from 1% to 1.75% (*w/w*). This base was used to test a range of concentrations of sodium bicarbonate as active pharmaceutical ingredients. Preservatives were not included at this stage to decrease the risk of contributions to vaginal toxicity. Microbial preservation may be assured by sodium bicarbonate itself or may not be needed if the product is marketed in individual doses (3 g applicator dose).

#### 3.1.1. Sensorial Properties, Osmolality and pH

As expected, sodium bicarbonate increased the pH value of the base formulation. All gels presented alkaline pH values, although no relevant changes were observed for pH in relation to the concentration of sodium bicarbonate in the tested range, ranging between 9.06 for the 4% (*w/w*) sodium bicarbonate gel and 9.02 at 25.2 °C for the 7% (*w/v*) sodium bicarbonate gel. pH measurements performed at 37 °C presented similar or slightly higher values, ranging from 9.11 to 9.09 for 4% and 7% (*w/w*) sodium bicarbonate gels (Table 3). Interestingly, dilutions with acidic vaginal fluids did not significantly reduce the pH values of the mixture (in some cases it slightly increased). It would be expected that an intermediate pH value between that of the undiluted gels and 3.5 would be achieved, as observed for the base formulation. These results are likely related to the low buffer capacity of the simulated vaginal fluid associated with the high concentration of sodium bicarbonate needed to neutralize an acidic pH and still be available in the medium [15,28].

Osmolality was increased with the bicarbonate concentration since the polymer did not significantly contribute to such parameter, as shown by the base (undosed) gel (Table 3). Even so, the values obtained for gels up to 6% concentration were below the higher limit recommended for lubricants (1200 mOsmol/Kg) [27,39]. Upon dilution with vaginal fluid in simulated conditions of cytolytic vaginosis, a decrease in osmolality was observed for all gels. This was explained by the dilution effect, with values of less than 1000 mOsm/Kg for gels up to 6% (*w/w*) sodium bicarbonate concentration. These results are in accordance with our previous observations for HPMC-based gels [15]. 

#### 3.1.2. Viscosity

**Figure 1:**
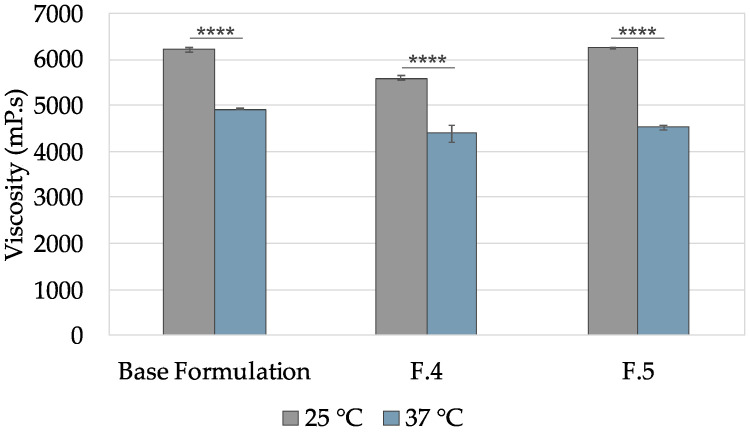
Determination of viscosity directly at room temperature (25 °C) and at physiologic temperature 37 °C. Gels F4 and F5 were determined using the shear rate 20 1/s. Results are presented as the mean ± SD, and n = 3. **** *p* < 0.0001.

The concentration of sodium bicarbonate influenced the viscosity of the gels. with F5 being more viscous and F7 less viscous at room temperature. Interestingly, when heated to physiologic temperature, the gels with the higher concentration of sodium bicarbonate were destabilized, and it was observed that phase separation occurred on the plate, probably due to the precipitation of sodium bicarbonate. Viscosity measurements were not considered valid for these conditions since a homogeneous preparation was not encountered on the plate after the test. This loss of viscosity may limit the application of such products. Heating F4 and F5 formulations decreased their viscosity, as observed for the base formulation (Figure 1). 

Formulations F4 and F5 were further tested for viscosity at 37 °C after dilution with mVFS (pH 3.5). In this specific condition, both gels suffered a dramatic loss of viscosity, which could no longer be measured at the same shear rate (torque was less than 10%). When the shear rate was increased to 300 s^−1^, F4 had a viscosity of 196 ± 11 and F5 a viscosity of 114 ± 5. This change in viscosity was due to the dilution effect with vaginal fluids and could influence the retention of such a formulation in the vaginal cavity. 

#### 3.1.3. Preliminary Stability Results 

To assess the stability of these gels under different storage conditions, a first worst-case scenario was tested by using the more concentrated gel (F7) and by stressing the product to extreme temperatures. The gel lost its original viscosity during the test and changed to a liquid formulation (Table 4). This result showed that the gel is extremely sensitive to high temperatures and further supports the observations of viscosity measurements under 37 °C. Gels with lower dosages of sodium bicarbonate (F4, F5, and F6) were stored for 1 months at 5 °C and kept their original properties, unlike those stored at room temperature (F5, F6, and F7). A white precipitate was observed at room temperature for concentrations higher than 5% (*w/w*). So, refrigeration was defined as mandatory for storage of this product. 

**Figure 2:**
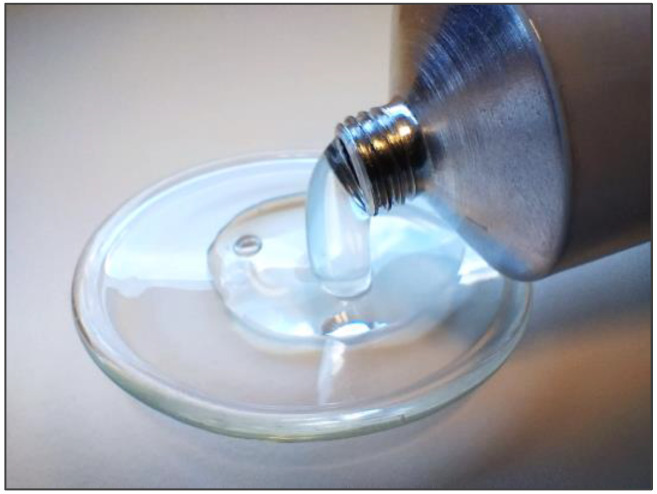
Macroscopic appearance of the 4% (*w/w*) sodium bicarbonate gel.

Figure 2 shows the macroscopic appearance of the 4% (*w/w*) sodium bicarbonate gel formulation, highlighting important characteristics such as its semi-solid consistency, fluidity, homogeneity, colourlessness, and transparency.

### 3.2. Antimicrobial Susceptibility Testing

#### 3.2.1. Minimal Inhibitory Concentration (MIC)

The minimal inhibitory concentration (MIC) was determined in *L. crispatus* vaginal isolates collected from healthy women and women diagnosed with cytolytic vaginosis. The broth microdilution method allowed for the determination of the lowest concentration at which sodium bicarbonate solution and gels with sodium bicarbonate inhibited the microbial growth of *L. crispatus* strains. Table 5 presents the visual MIC values determined after 24 h of incubation.

Sodium bicarbonate solution showed MIC values ranging from 3.13 to 12.50 mg/mL. *L. crispatus* PB7 was the most susceptible, with an MIC value of 3.13 mg/mL. *L. crispatus* LF7, LF9, and PB10 were the least susceptible strains, with MIC value of 12.50 mg/mL. 

For the sodium bicarbonate gels, all strains were shown to be susceptible to at least 25% (*v/v*) dilution of the products. Small variations in the results occurred, with some dependent on the testing strains and others on the concentration of sodium bicarbonate in the gel. *L. crispatus* PB7 was the most sensitive strain to all gels tested, with an MIC value of 6.25% (*v/v*) dilution. With a similar profile to the sodium bicarbonate solution, *L. crispatus* LF7, LF9, and PB10 showed lower susceptibility to the gels with sodium bicarbonate. The gel base (B—without sodium bicarbonate) showed no antimicrobial activity.

**Figure 3:**
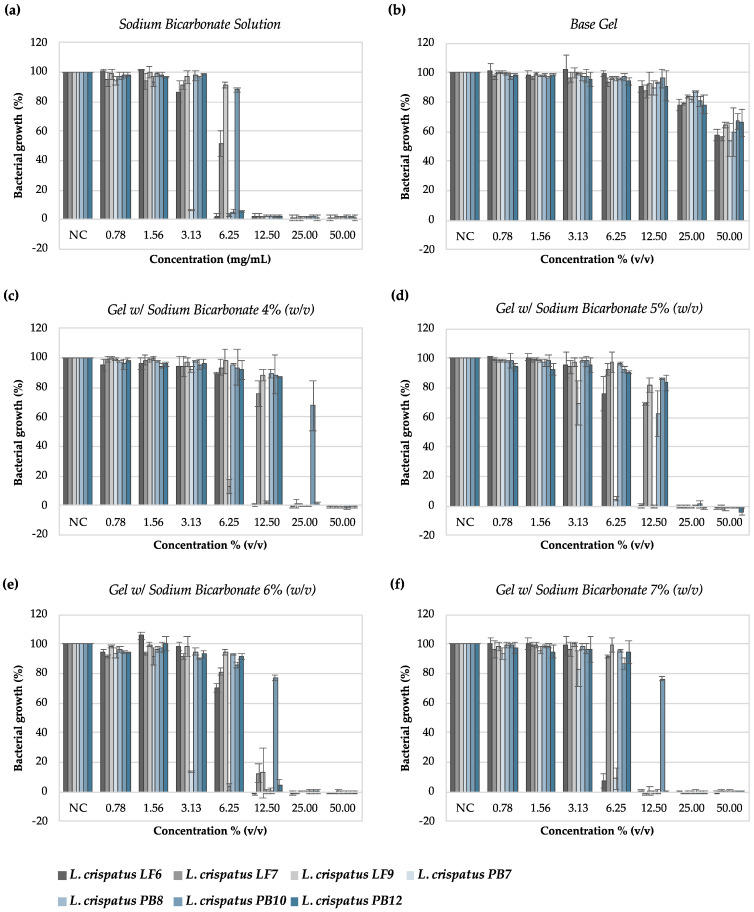
*Lactobacillus crispatus* growth measured using optical absorbance after treatment with sodium bicarbonate solution, gel base, and gels with sodium bicarbonate. (**a**) Sodium bicarbonate solution; (**b**) gel base; (**c**) gel with 4% (*w/w*) sodium bicarbonate; (**d**) gel with 5% (*w/w*) sodium bicarbonate; (**e**) gel with 6% (*w/w*) sodium bicarbonate; (**f**) gel with 7% (*w/w*) sodium bicarbonate. NC—negative control. Results are presented as the mean ± SD, and n = 3. Grey bars—*Lactobacillus crispatus* isolated from healthy women; Blue bars—*Lactobacillus crispatus* isolated from women diagnosed with cytolytic vaginosis.

Figure 3 shows the growth profiles of the *L. crispatus* strains in contact with the sodium bicarbonate solution, gel base, and gels with sodium bicarbonate.

In accordance with the visual MIC values, the optical measurements of the bacterial growth after treatment with the testing samples evidenced that the bicarbonate solution prevented the bacterial growth at concentrations ≥12.50 mg/mL. The lowest MIC_50_ value obtained was 2.22 mg/mL. This was for *L. crispatus* PB7, suggesting that this strain is more sensitive to sodium bicarbonate than others. On the other hand, although it was not possible to determine the visual MIC for the gel base (Table 6), one could confirm that it impaired the growth of *L. crispatus* strains, with an MIC_50_ mean value of 63.18 ± 6.69% (*v/v*).

*L. crispatus* PB10 was the least susceptible strain to both the solution and the gels with sodium bicarbonate, showing higher MIC_50_ and MIC_90_ values than the other strains.

The gel with 6 and 7% (*w/w*) sodium bicarbonate caused an evident reduction in the growth of all *L. crispatus* strains. However, the gels with 4 and 5% (*w/w*) sodium bicarbonate showed similar effects for dilutions equal to 50% (*v/v*). 

#### 3.2.2. Minimal Bactericidal Concentration (MBC)

The minimal bactericidal concentrations (MBCs) of the sodium bicarbonate solution, gel base, and the gels with sodium bicarbonate in *L. crispatus* strains are shown in Table 7.

It was possible to determine the MBC for all gels and for the solution with sodium bicarbonate. The MBC values ranged from 6.25 to 25.00 mg/mL for the sodium bicarbonate solution and from 6.25 to 50.00% (*v/v*) dilutions for the gels. The gel base showed no bactericidal activity on tested *L. crispatus* strains, with an MBC value > 50.00% (*v/v*) dilution.

The dose-dependent effect of sodium bicarbonate in the gels generally emphasizes the cidal effect on the strains tested. Additionally, the MBC values (6.25% (*v/v*) dilution) obtained for the sodium bicarbonate gels suggest that there is a bactericidal effect on *L. crispatus* PB7. Even though the gels show an apparent bacteriostatic effect for the remaining strains, at 50% (*v/v*) dilution, they completely reduce bacterial growth (with an absence of CFU). 

#### 3.2.3. Effect of pH on *Lactobacillus crispatus* Growth

**Figure 4:**
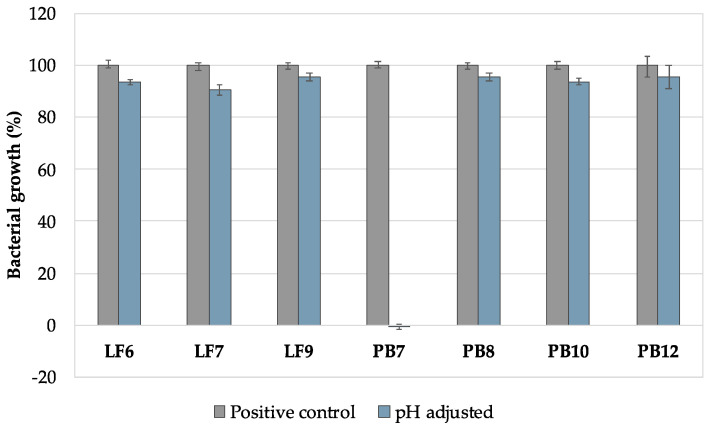
Bacterial growth of *Lactobacillus crispatus* strains after contact with the pH-adjusted (alkalinized) culture medium. Results are presented as the mean ± SD, and n = 3.

In order to evaluate the mechanism of action of sodium bicarbonate, and since it strongly alkalinizes the culture medium used in the antimicrobial activity assays, studies were performed in the presence of a culture medium adjusted to pH 8.6. Figure 4 shows the growth profile of the strains after exposure to the pH-adjusted culture medium.

All strains showed a survival rate higher than 90% when compared to the growth control (culture medium at pH 6.5), except for *L. crispatus* PB7. This strain showed a pronounced intolerance to pH change (alkaline environment), indicating that the results obtained for the formulations with sodium bicarbonate were due not only to its presence but also to the pH alkalinization.

### 3.3. In Vitro Cytotoxicity Testing

#### 3.3.1. Cellular Cytotoxicity

The formulations’ cytotoxicity was tested in different cell lines (Hec-1A, HeLa, and VK2 E6/E7) according to the ISO 10993-5 standard [34]. The concentrations tested were the same as for antimicrobial susceptibility testing and ranged from 0.78 to 50 mg/mL for the sodium bicarbonate solution and from 0.78 to 50% (*v/v*) dilutions for the gel base and gels with sodium bicarbonate. 

**Figure 5:**
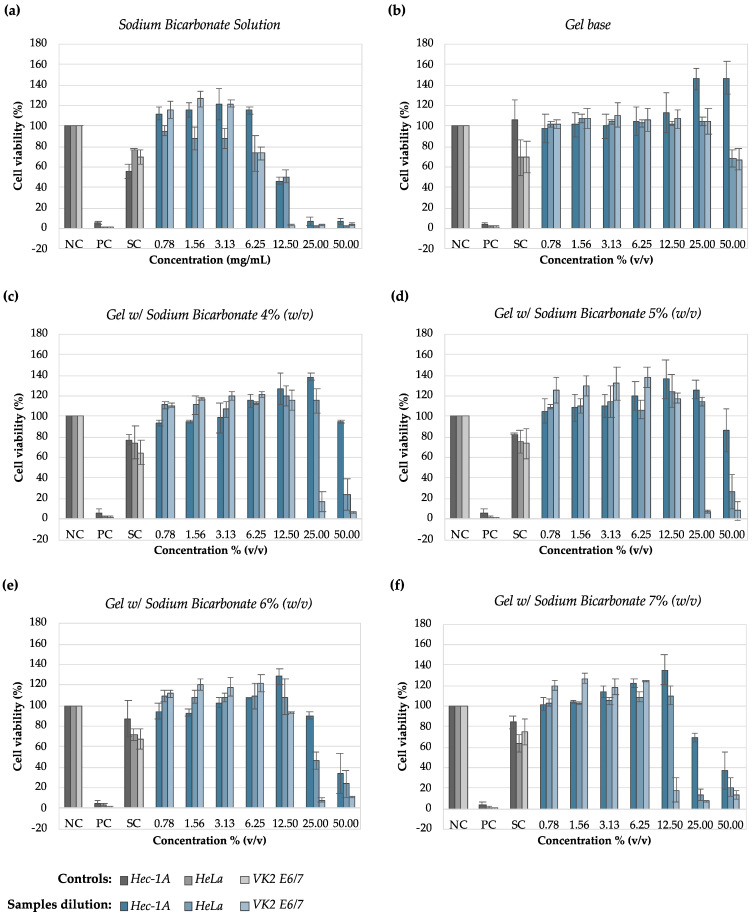
Cytotoxic profiles of sodium bicarbonate solution, gel base, and gel with sodium bicarbonate in Hec-1A, HeLa, and VK2 E6/E7 cell lines. (**a**) Sodium bicarbonate solution; (**b**) gel base; (**c**) gel with 4% (*w/w*) sodium bicarbonate; (**d**) gel with 5% (*w/w*) sodium bicarbonate; (**e**) gel with 6% (*w/w*) sodium bicarbonate; (**f**) gel with 7% (*w/w*) sodium bicarbonate. Results are presented as the mean ± SD, and n = 6. Grey bars—experimental controls: NC—negative control; PC—positive control; SC—solvent control; Blue bars—dilutions of test samples.

Figure 5 shows the cytotoxicity results for the three cell lines. Grey bars represent the controls (negative, positive, and solvent control), and the blue bars represent the dilutions performed on test samples in the different cell lines. According to ISO 10993-5, test sample concentrations that affect growth by more than 30% (70% cell viability) are considered cytotoxic.

Regarding the sodium bicarbonate solution, concentrations from 12.00 to 50.00 mg/mL were cytotoxic in all three cell lines, with reductions in cell viability of more than 30%. The effect was most evident at 25.00 and 50.00 mg/mL concentrations, with a cell viability reduction greater than 90%. The CC_50_ (Table 8) for the sodium bicarbonate solution for the Hec-1A, HeLa, and VK2 E6/E7 lines was 12.49, 11.70, and 7.71 mg/mL, respectively. However, the results obtained for the sodium bicarbonate solution from 0.78 to 3.13 mg/mL suggest positive interference (approximately 20%) with the cellular growth of Hec-1 and VK2 E6/E7 cell lines.

The gel base was not cytotoxic at any of the concentrations tested (0.78 to 50% (*v/v*) dilutions), despite the slight reduction in cell viability observed in HeLa and VK2 E6/E7 lines for the highest concentration tested. The CC_50_ values, calculated by extrapolation (Table 8), for HeLa and VK 2 E6/E7 were 55.78 and 56.43% (*v/v*), respectively. In contrast, the gel base showed a positive influence on the cell growth of the Hec-1A line when dilutions of 25 and 50% (*v/v*) were tested as an increase in cell growth of more than 50% was observed compared to the control. It was not possible to determine CC_50_.

Gels with 4 and 5% (*w/w*) sodium bicarbonate showed similar degrees of cytotoxicity on Hec-1A, HeLa, and VK2 E6/E7 cell lines. The 25.00% (*v/v*) dilution of both gels was cytotoxic in the VK2 E6/E7 cell line, and the 50% (*v/v*) dilution was cytotoxic in HeLa and VK2 E6/E7 cell lines. The 4 and 5% (*w/w*) bicarbonate gels showed no cytotoxic effect (cell viability higher than 70%) on the Hec-1A cell line. Gel dilutions between 0.78 and 25.00% (*v/v*) appeared to interfere positively with cellular growth. The CC_50_ values for all gels are shown in Table 8.

Gels with 6 and 7% (*w/w*) of sodium bicarbonate were cytotoxic in all tested cell lines, as the 50% (*v/v*) dilution reduced cell viability by more than 30%. Similar to the gels with 4 and 5% (*m/v*) sodium bicarbonate, the Hec-1A cell line seems to be the least susceptible to sodium bicarbonate gels since it shows higher cell viability values (CC_50_: 14.33 and 9.51% (*v/v*) for HeLa and VK2 E6/E7, respectively). In contrast, the VK2 E6/E7 cell line is the most susceptible to all the formulations tested. However, intermediate dilutions of these gels (1.56 to 12.50 mg/mL) seem to interfere positively with cellular growth.

#### 3.3.2. Effect of pH on the Viability of Cell Lines

**Figure 6:**
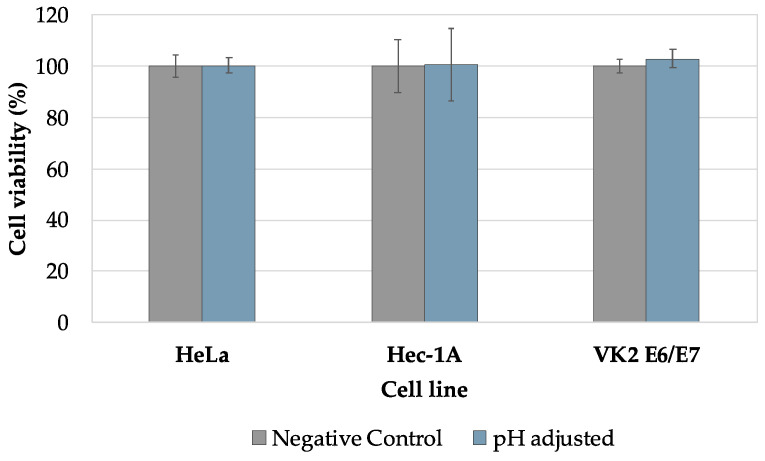
Cellular viability of HeLa, Hec-1A, and VK2 E6/E7 after incubation with alkalinized culture medium (pH 8.3). Results are presented as the mean ± SD, and n = 3.

Figure 6 provides evidence that the effect on cellular cytotoxicity was not caused by the culture medium’s alkalinization but by the sodium bicarbonate’s chemical nature.

None of the cell lines (HeLa, Hec-1A and VK2 E6/E7) was susceptible to pH variation since cellular behaviour was similar to that of the negative control (culture medium with pH 7.4).

### 3.4. In Vitro Interference of Lactobacillus crispatus Adhesion to Epithelial Cells

The interference of the gel containing 4% (*w/w*) sodium bicarbonate with the adhesion of *L. crispatus* PB8 to the epithelial cells was studied in three cell lines: Hec-1A, Hela, and VK2 E6/E7. This strain was selected for its strong ability to adhere to the three selected cell lines. Gel with 4% (*w/w*) sodium bicarbonate was selected based on its antimicrobial susceptibility and cytotoxicity testing results. Three different approaches were used: competition, exclusion, and displacement.

**Figure 7:**
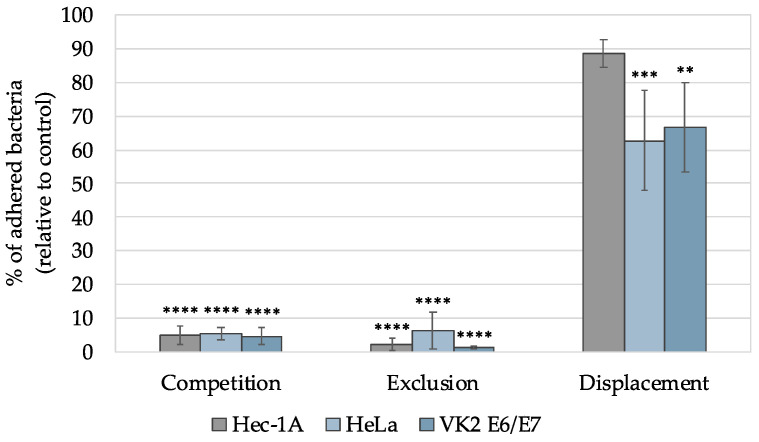
Adhesion profiles of the *Lactobacillus crispatus* PB8 strain to Hec-1A, HeLa, and VK2 E6/E7 cell lines in the presence of the gel with sodium bicarbonate 4% (*m/v*). Results are presented as the mean ± SD, and n = 3. ** *p* < 0.01, *** *p* < 0.001, and **** *p* < 0.0001 represent statistical significance in comparison with the control (100% bacterial adhesion).

Figure 7 shows the results obtained for each approach applied to the three cell lines.

**Figure 8:**
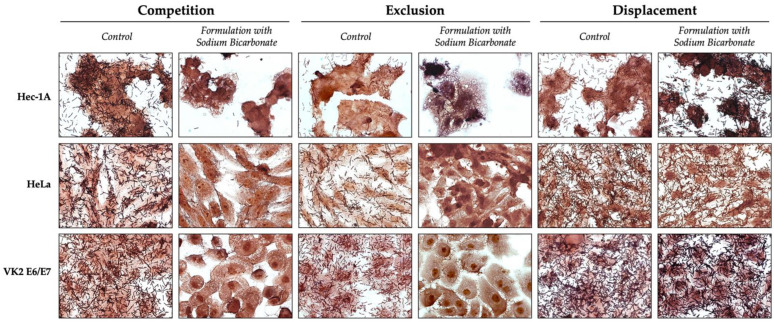
Representative microphotographs (630×) of the different approaches used to study the interference of the gel containing 4% (*w/w*) sodium bicarbonate with *Lactobacilli crispatus* PB8 adhesion to epithelial cells. Each row of the panel corresponds to a cell line (Hec-1A, HeLa, and VK2 E6/E7), and in each column, the different approaches (competition, exclusion, and displacement) that were performed for the control and the gel with 4% (*w/w*) sodium bicarbonate are shown.

The adhesion profile was similar in all three cell lines and for all three approaches studied. Regarding competition, the gel with sodium bicarbonate 4% (*w/w*) was placed, simultaneously with the *L. crispatus* PB8, in contact with the cells, and it was possible to observe that the gel significantly prevented adhesion (*p* < 0.0001) by more than 90%. The percentage of bacteria that adhered to Hec-1A, HeLa, and VK2 E6/E7 lines was 4.98 ± 2.68, 5.41 ± 1.96, and 4.57 ± 2–60, respectively. Also, in Figure 8 (competition panel), it is possible to observe that the experimental control shows the dominance of *Lactobacillus,* in terms of adhering to the cells, which were removed by exposure to the gel with sodium bicarbonate.

In the exclusion approach, a gel with sodium bicarbonate 4% (*w/w*) was placed in contact with the cell lines, followed by infection with the *L. crispatus* PB8 strain. This approach was intended to evaluate the effect of the gel in terms of blocking the adhesion sites of epithelial cells. Similar to the competition approach, it was found that the gel significantly prevented (*p* < 0.0001) adhesion as more than 90% of the *L. crispatus* PB8 were not able to adhere to the epithelial cells. The effect of the gel was more pronounced on the Hec-1A (2.18 ± 1.88% of adhered bacteria) and VK2 E6/E7 (1.37 ± 0.41% of adhered bacteria) lines than on the HeLa (6.35 ± 5.41% of adhered bacteria) line. Figure 8 (exclusion panel) shows the absence of *L. crispatus* PB8 in the cells treated with gel with 4% (*w/w*) sodium bicarbonate. 

The displacement approach evaluated the ability of the gel containing sodium bicarbonate 4% (*w/w*) to remove or displace the *L. crispatus* PB8 from the epithelial cells. This approach was demonstrated to be the least effective. The percentage of *L. crispatus* PB8 when it adhered to Hec-1A, HeLa, and VK2 E6/E7 was 88.59 ± 4.07, 62.72 ± 14.86 and 66.74 ± 13.42, respectively. Thus, *L. crispatus* PB8 strain was significantly removed (*p* < 0.001) from HeLa and VK2 E6/E7 cells, but not from the Hec-1A cell line (*p* > 0.05). Also, reviewing the results in Figure 8, it is possible to verify the presence of *Lactobacillus* in both control and gel-treated cells.

### 3.5. Tests in Vaginal Explants Tissues

**Figure 9:**
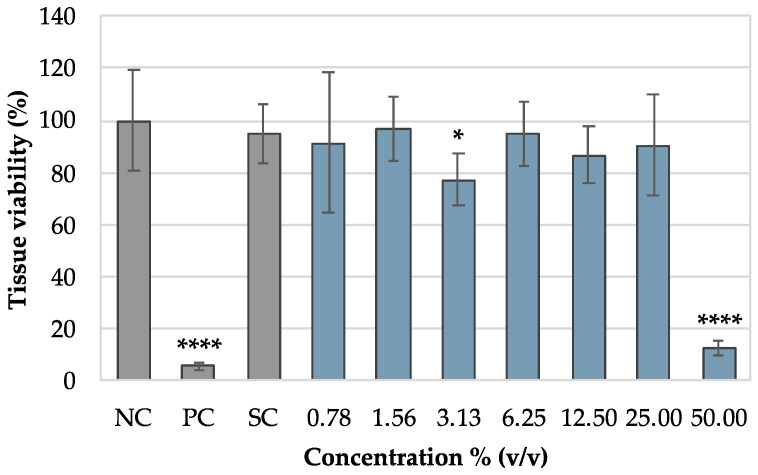
Tissue viability profile for gel with 4% (*w/w*) sodium bicarbonate at dilutions ranging from 0.78 to 50% (*v/v*). NC—negative control; PC—positive control; SC—solvent control. Results are presented as the mean ± SD, and n = 8. * *p* < 0.05; **** *p* < 0.0001 represents statistical significance in comparison with negative control.

A vaginal porcine ex vivo model [35] was selected in order to evaluate the toxicity in a model more representative of the human vagina. Gel with 4% (*w/w*) sodium bicarbonate was selected according to the experimental results obtained in the antimicrobial susceptibility testing and in vitro cytotoxicity assays. Figure 9 shows the viability of explants after exposure to the gel with 4% (*w/w*) sodium bicarbonate. The negative control (culture medium), positive control (5% SDS (*v/v*)), and solvent control (50% (*v/v*)-diluted culture medium) were used to evaluate the response of this model to the test sample.

A 50% (*v/v*) dilution of the sodium bicarbonate gel decreased explant viability significantly (*p* < 0.0001) by more than 80% compared to the negative control (culture medium). In this model, the CC_50_ value was 45.47% (*v/v*) for the gel with 4% (*w/w*) sodium bicarbonate. As expected, the positive control (SDS 5% (*v/v*)) showed a significant reduction (*p* < 0.0001) in explant viability of more than 90%. This result demonstrates that the selected model is responsive to the test samples under study. 

## 4. Discussion

Vaginal gels were developed with sodium bicarbonate for CV to improve therapeutic efficacy while complying with women’s preferences for vaginal dosage forms [40,41]. As expected, based on the physicochemical properties of the active substance, all gels presented alkaline pH values, either when measured at 25 °C or at body temperature (37 ± 0.2 °C). The alkaline pH of the gels will be decisive for therapeutic success, since the alkalinization of the vaginal environment is the empirical treatment traditionally used for cytolytic vaginosis. In fact, the traditional treatment of cytolytic vaginosis consists of sitz baths with sodium bicarbonate solutions (30 to 60 g of sodium bicarbonate to approximately 1 L of water) two to three times per week [1,13]. When mixed with acidic simulated vaginal fluids, the overall pH of the mixture was still alkaline, indicating the ability of the gel to change the acidity of the vaginal environment. Results obtained with the less dosed gel (4% (*w/w*)) were not less alkaline than those obtained with higher concentrations of sodium bicarbonate. We have previously assessed the pH of sodium bicarbonate solutions traditionally prescribed as treatment for CV as 8.18 for the 3% sodium bicarbonate solution and 8.51 for the 6% (*w/w*) sodium bicarbonate solution [15]. So, while being clearly outside the pH range advised for vaginal products intended for long-term use [39], the pH values of the gels and their mixtures with fluids are basic pH values. This similar to the currently used liquid approach to treating CV. The results obtained in this study are in accordance with our previous results for a formulation containing HPMC 1% (*w/w*) and sodium bicarbonate at 5% (*w/w*) in terms of pH [15]. Also, the osmolality results of the 5% (*w/w*) formulations (F5 of the present study and D of the previous work) were concordant [15]. In this study, we explored the possibility of decreasing the sodium bicarbonate concentration to 4% (*w/w*). While the pH did not significantly decrease, the osmolality of this gel was lower and further decreased upon dilution with vaginal fluids to values lower than the maximum limits recommended for lubricants by WHO (1200 mOsm/Kg) [39]. Since osmolality has been correlated with vaginal irritation, this decrease in concentration might translate to increased safety. While concerns may arise due to the alkaline pH values obtained for such gels, we decided to explore the concentrations of sodium bicarbonate in the range of 4% to 7% (*w/w*) (and not lower than 4% (*w/w*)) based on reports of a loss of efficacy and even the promotion of virulence factors by the use of low concentrations of sodium bicarbonate [42,43,44]. Nevertheless, it is important to consider the risk of using alkaline products for vaginal application, especially if the envisaged application is not clearly designated for a time-limited period [39]. 

Compared to the sodium bicarbonate solution, which is the traditional treatment of cytolytic vaginosis, the sodium bicarbonate gels naturally presented higher viscosity levels. These results suggest that they are less likely to leak through the vaginal canal, presenting longer retention time in the vagina. Unlike irrigation with sodium bicarbonate solutions, these sodium bicarbonate gels will allow prolonged and intimate contact with the vaginal epithelium to promote therapeutic success. For comfort upon application and retention, it is important to consider the performance of the gel upon dilution with vaginal fluids (or surrogates) [41]. Our results from all tests show that bicarbonate gels based on HPMC are extremely sensitive to temperature (either when tested for viscosity or in preliminary stability tests). This polymer was selected due to its nonionic nature and known resistance to salts superior to other commonly used bioadhesive polymers (such as carbomers) [45]. While the sol–gel transition of HPMC is mainly triggered by temperature, these gels are known to be sensitive to extreme pH environments [45], so combinations of both factors may influence viscosity and the overall performance of these products. HPMC was selected as the viscosity agent for these gels based on our previous results, which showed that when viscosity was low (improved in the present study by increasing the concentration of the polymer), an interesting bioadhesive profile of this base could be observed. In this study, the sodium bicarbonate concentration clearly affected the viscosity of the gel, as shown with formulations above 6% (*w/w*), which resulted in gels that dramatically lose viscosity and change structure when heated at body temperature (although results at room temperature may be acceptable). These results further stress the importance of considering physiologic and pathological surrogates during the in vitro testing of prototypes for vaginal applications, as previously discussed [28]. Only formulations F4 and F5 resisted heating at 37 °C, although both lost viscosity when diluted and heated simultaneously. Dilution in vaginal fluids at body temperature is indeed critical for efficacy and comfort. Adjusting these formulations with polymers less sensitive to these factors and to the sodium bicarbonate concentration should be considered in future research [28,46]. 

The antimicrobial activity [33] was tested considering the MIC determination for both the developed formulations and the sodium bicarbonate solution against 7 *L. crispatus* strains, isolated from healthy women (n = 3) and women with cytolytic vaginosis (n = 4). Indeed, the sodium bicarbonate solution and the gels with different sodium bicarbonate concentrations (tested at a maximum dilution of 50% (*v/v*)) effectively inhibited the bacterial growth of the *L. crispatus* strains. As expected, the base gel without sodium bicarbonate was inactive against the tested strains, although a slight negative interference with growth was observed. These results demonstrate the efficacy of all gels on the *L. crispatus* strains, even the gel with the lowest sodium bicarbonate concentration (4% (*w/w*)). Additionally, besides inhibiting the growth of *L. crispatus*, all gels could eliminate this species (the absence of CFUs) efficiently. The same susceptibility profile was observed for all the enrolled strains, independently of their origin (healthy women and women diagnosed with cytolytic vaginosis). The mechanism of action needs to be further clarified. However, it has been found that the antimicrobial activity of sodium bicarbonate is related to its chemical nature and not only to the alkalinization of the culture medium. 

Cellular cytotoxicity was tested on three cell lines of the female reproductive system (Hec-1, HeLa, and VK2 E6/E7) according to the international standard ISO 10993-5 [34]. In general, increasing the concentration of sodium bicarbonate in the gels led to increased cellular cytotoxicity in the tested lines, with an evident reduction in cell viability (greater than 70%). Similar results were obtained for bicarbonate gels used to treat vulvovaginal candidiasis [15]. This result is corroborated by testing sodium bicarbonate solution alone at higher dilutions (from 12.50 to 50.00 mg/mL). Although the experimental results demonstrate cellular cytotoxicity, it is known that monolayer cell models are susceptible to damage caused by the direct contact of samples [47]. However, these models are very useful for screening and comparing the characteristics of different formulations under the same experimental conditions. The gels with 4% and 5% (*w/w*) sodium bicarbonate were demonstrated to be more compatible. Furthermore, the gel base (HPMC-based) and intermediate dilutions of sodium bicarbonate solution (0.78 to 6.25 mg/mL) positively affected cell proliferation. This result may be induced by the HPMC present in the gel base formulation as some authors refer to its influence on cell growth [48]. Thus, gels of 4 and 5% (*w/w*) concentrations were shown to be the most promising since they demonstrated less cellular cytotoxicity but still retained antimicrobial activity against *L. crispatus* strains. Similar to the results obtained for antimicrobial activity, pH also seems not to influence cell viability. 

To overcome the limitations of unicellular models, an ex vivo assay was performed on the previously described vaginal porcine model [35]. The 50% (*v/v*) dilution of the gel with 4% (*w/w*) sodium bicarbonate demonstrated histotoxicity in vaginal tissue, but for dilutions of 25% (*v/v*) and lower no toxicity was observed. These results are in accordance with other formulations for vaginal application that were previously tested by our research group [35]. In the previous work, one could observe that products that are commercially available and validated for vaginal application present histotoxicity for dilutions of 20% (*v/v*). The 4% (*w/w*) sodium bicarbonate gel, in contrast, was observed to be non-toxic at 25% (*v/v*) [35]. This result demonstrates that although the gels show toxic effects in unicellular and ex vivo models, their safety is not compromised when compared to products already available for vaginal application. 

We performed interference assays using the sodium bicarbonate gel 4% (*w/w*) on competition, exclusion and displacement were performed of *L. crispatus* PB8. The sodium bicarbonate gel with 4% (*w/w*) showed high efficiency when competing with the binding sites of the *L. crispatus* PB8 to epithelial cells. Additionally, this gel can block these same adhesion sites, preventing the strain from binding to cell lines. The gel showed the worst ability in terms of displacement, where the reduction in *L. crispatus* PB8 was not more than 30%. However, in the HeLa and VK 2 E6/E7 cell lines, it showed a significant reduction compared to the control. These tests were used considering the strategy described for probiotic products efficacy evaluation [49,50].

As the symptoms related to CV are consequences of the pathogenic effect of *L. crispatus*, we designed a study that, besides evaluating the antimicrobial activity of the proposed products, could also elucidate their effect in the prevention of bacterial adhesion to cells. Until now, the in vitro antimicrobial activity of this molecule had not yet been evidenced in this *L. crispatus* species. For Candida genus, the antimicrobial effect of sodium bicarbonate gels was previously described [15,51,52]. In fact, the use of sodium bicarbonate in douching or as suppository has been suggested as a strategy for the treatment of cytolytic vaginosis [1,13,14]. However, it is thought that the high concentrations of lactic acid produced by the genus Lactobacillus may be one of the factors leading to the burning sensation and damage of vaginal epithelium caused by the lysis of epithelial cells [1,53]. The alkalinization of the vaginal microenvironment and consequent neutralization of the acids produced by the Lactobacillus species may occur through sodium bicarbonate molecules. This is already used in the treatment of other pathologies related to hyperacidity or electrolyte imbalance [16,17,18,19,20]. 

Additionally, the gel with sodium bicarbonate 4% (*w/w*) demonstrated biocompatibility comparable to other products already available on the market, as well as physicochemical characteristics that will allow it to deliver the sodium bicarbonate as an active molecule into the vaginal microenvironment for short treatment courses. Nevertheless, negative effects of prolonged use of alkaline products in the vagina should not be underestimated and further studies should be performed to assess potential negative impacts in such cases. 

## 5. Conclusions

This study allowed the development of different prototype gels with increasing concentrations of sodium bicarbonate to reverse or at least alleviate symptoms in women with cytolytic vaginosis. Efficacy was demonstrated for the first time in treating *L. crispatus* isolated from women with cytolytic vaginosis, and the product was safe in vitro and ex vivo. Gel with sodium bicarbonate 4% (*w/w*) was demonstrated to be the prototype with the most suitable physicochemical, antibacterial, and biocompatibility characteristics for vaginal application in the treatment of cytolytic vaginosis. However, although the in vitro and ex vivo results provide valuable insights into the gel’s compatibility and efficacy, clinical trials will be necessary to confirm its safety and therapeutic effectiveness in use conditions. Furthermore, its interactions with other microorganisms in the vaginal microbiome and its potential impacts on other *Lactobacillus* species were not evaluated. For a more comprehensive evaluation of stability, future studies will follow ICH guidelines, which will allow for longer-term assessments and provide more robust data on the formulation’s stability over time. 

## Figures and Tables

**Table 1:** Qualitative and quantitative (% *w/w*) composition of the gels.

Components	Base Formulation	F4	F5	F6	F7
Sodium bicarbonate	0	4.00	5.00	6.00	7.00
HPMC K100M	1.75	1.75	1.75	1.75	1.75
Water	98.25	94.25	93.25	92.25	91.25

**Table 2:** Strains used in the study and their clinical group.

Strain	Clinical Group
*Lactobacillus crispatus* LF6	Healthy
*Lactobacillus crispatus* LF7	Healthy
*Lactobacillus crispatus* LF9	Healthy
*Lactobacillus crispatus* PB7	Cytolytic vaginosis
*Lactobacillus crispatus* PB8	Cytolytic vaginosis
*Lactobacillus crispatus* PB10	Cytolytic vaginosis
*Lactobacillus crispatus* PB12	Cytolytic vaginosis

**Table 3:** Sensorial properties and pH values as measured directly on the gel base and gels with 4 to 7% (*w/w*) sodium bicarbonate.

	Sensorial Properties	Osmolality (mOsm/Kg)		pH Direct Measurements		pH Diluted mVFS	
		Direct	Dilution mVFS	(25 ± 0.2 °C)	(37 ± 0.2 °C)	(25 ± 0.2 °C)	(37 ± 0.2 °C)
Base gel	Semi-solid. Very viscous, consistent gel. Homogenous. Colourless, transparent. Odourless. Soft.	19 ± 2	67 ± 3	5.75 ± 0.01	6.52 ± 0.12	3.64 ± 0.03	3.60 ± 0.05
F4	Semi-solid. Fluid gel. Homogenous. Colourless, transparent. Odourless.	839 ± 3	706 ± 7	9.06 ± 0.00	9.11 ± 0.01	9.00 ± 0.05	9.05 ± 0.04
F5	Semi-solid. Fluid gel. Homogenous. Colourless, transparent. Odourless.	1050 ± 4	862 ± 2	8.93 ± 0.01	8.99 ± 0.01	8.84 ± 0.03	8.98 ± 0.06
F6	Semi-solid. Very viscous, consistent gel. Homogenous. Colourless, transparent. Odourless. Soft, not particulated.	1189 ± 4	963 ± 10	8.83 ± 0.02	8.92 ± 0.03	8.98 ± 0.01	9.16 ± 0.11
F7	Semi-solid. Very viscous, consistent gel. Homogenous. Colourless, transparent. Odourless. Soft, not particulated.	1417 ± 7	1065 ± 4	9.02 ± 0.01	9.09 ± 0.01	9.11 ± 0.08	9.31 ± 0.15

**Table 4:** Sensorial parameters of sodium bicarbonate-based gels after preparation (t0) and after 4 weeks in glass packaging maintained at room temperature (20–25 °C), refrigerated (5 °C), or under a cycling temperature (5 °C or 40 °C each 24 h).

	Sensorial Properties	Temperature Cycles (4 Weeks)	RT (1 Month)	5 °C (1 Month)
F4	Semi-solid. Fluid gel. Homogenous. Colourless, transparent. Odourless.	ND	The gel remained homogeneous.	The formula maintained the same sensorial properties as t0.
F5	Semi-solid. Fluid gel. Homogenous. Colourless, transparent. Odourless.	ND	The gel lost viscosity. A white sediment was observed.	The formula maintained the same sensorial properties as t0.
F6	Semi-solid. Very viscous, consistent gel. Homogenous. Colourless, transparent. Odourless. Soft, not particulated.	ND	The gel lost viscosity. A white sediment was observed.	The formula maintained the same sensorial properties as t0.
F7	Semi-solid. Very viscous, consistent gel. Homogenous. Colourless, transparent. Odourless. Soft, not particulated.	After temperature cycles, the gel lost all viscosity, becoming a slightly turbid liquid.	The gel lost all viscosity, becoming a liquid. A white sediment was observed.	The gel lost all viscosity becoming liquid. A white sediment was observed.

ND—not determined.

**Table 5:** MIC values for the *Lactobacillus crispatus* strains after contact with sodium bicarbonate solution, gel base, and 4 to 7% (*w/w*) sodium bicarbonate gels, as defined by visual inspection of the plates.

Strain	S.B. (mg/mL)	F4 % (*v/v*)	F5 % (*v/v*)	F6 % (*v/v*)	F7 % (*v/v*)	B. % (*v/v*)
*L. crispatus* LF6	6.25	12.50	12.50	12.50	6.25	>50.00
*L. crispatus* LF7	12.50	25.00	25.00	25.00	12.50	>50.00
*L. crispatus* LF9	12.50	25.00	25.00	25.00	12.50	>50.00
*L. crispatus* PB7	3.13	6.25	6.25	6.25	6.25	>50.00
*L. crispatus* PB8	6.25	25.00	25.00	12.50	12.50	>50.00
*L. crispatus* PB10	12.50	25.00	25.00	25.00	12.50	>50.00
*L. crispatus* PB12	6.25	25.00	25.00	12.50	12.50	>50.00

S.B.—sodium bicarbonate solution; F4 to F7—gels with the sodium bicarbonate concentration increasing from 4 to 7% (*w/w*), respectively; B.—gel base.

**Table 6:** MIC_50_ and MIC_90_ for the *Lactobacillus crispatus* strains after contact with sodium bicarbonate solution, gel base, and 4 to 7% (*w/w*) sodium bicarbonate gels.

	S.B. (mg/mL)		F4 % (*v/v*)		F5 % (*v/v*)		F6 % (*v/v*)		F7 % (*v/v*)		B. % (*v/v*)	
Strains	MIC_50_	MIC_90_	MIC_50_	MIC_90_	MIC_50_	MIC_90_	MIC_50_	MIC_90_	MIC_50_	MIC_90_	MIC_50_	MIC_90_
*L. crispatus* LF6	3.79	4.76	8.13	10.30	7.23	9.33	7.00	9.26	5.64	6.16	58.54 *	N.D.
*L. crispatus* LF7	6.29	7.89	14.30	18.11	13.83	17.87	7.76	10.26	6.93	7.57	55.06 *	N.D.
*L. crispatus* LF9	8.00	10.03	15.92	20.16	15.13	19.54	9.83	12.99	9.88	10.80	69.07 *	N.D.
*L. crispatus* PB7	2.22	2.79	5.08	6.43	3.47	4.48	2.46	3.25	3.34	3.65	55.77 *	N.D.
*L. crispatus* PB8	4.65	5.83	16.12	20.42	13.33	17.21	8.58	11.34	7.24	7.90	64.96 *	N.D.
*L. crispatus* PB10	7.68	9.64	27.19	34.43	15.90	20.54	14.65	19.37	13.12	14.33	71.71 *	N.D.
*L. crispatus* PB12	4.70	5.89	15.88	20.12	15.29	19.74	8.74	11.56	7.12	7.78	67.12 *	N.D.

S.B.—sodium bicarbonate solution; F4 to F7—gel with the sodium bicarbonate concentration increasing from 4 to 7% (*w/w*), respectively; B.—gel base; * MIC_50_ values calculated by extrapolation; N.D.—not determined.

**Table 7:** MBC values for the *Lactobacillus crispatus* strains after contact with sodium bicarbonate solution, gel base, and 4 to 7% (*w/w*) sodium bicarbonate gels.

Strain	S.B. (mg/mL)	F4 % (*v/v*)	F5 % (*v/v*)	F6 % (*v/v*)	F7 % (*v/v*)	B. % (*v/v*)
*L. crispatus* LF6	25.00	25.00	12.50	12.50	6.25	>50.00
*L. crispatus* LF7	25.00	50.00	50.00	25.00	25.00	>50.00
*L. crispatus* LF9	25.00	50.00	50.00	25.00	25.00	>50.00
*L. crispatus* PB7	6.25	6.25	6.25	6.25	6.25	>50.00
*L. crispatus* PB8	12.50	25.00	25.00	12.50	12.50	>50.00
*L. crispatus* PB10	25.00	50.00	50.00	25.00	25.00	>50.00
*L. crispatus* PB12	12.50	50.00	25.00	25.00	25.00	>50.00

S.B.—sodium bicarbonate solution; F4 to F7—gels with the sodium bicarbonate concentration increasing from 4 to 7% (*w/w*), respectively; B.—gel base.

**Table 8:** CC_50_ values for the Hec-1A, HeLa, and VK2 E6/E7 cell lines after contact with sodium bicarbonate solution, gel base, and gel with 4 to 7% (*w/w*) sodium bicarbonate.

Cell Line	S.B. (mg/mL)	F4 % (*v/v*)	F5 % (*v/v*)	F6 % (*v/v*)	F7 % (*v/v*)	B. % (*v/v*)
Hec-1A	12.49	N.D.	55.14 *	52.83 *	41.37	25.49
HeLa	11.70	55.72 *	47.05	47.90	24.50	14.33
VK2 E6/E7	7.71	55.43 *	22.85	18.50	17.12	9.51

S.B.—sodium bicarbonate solution; F4 to F7—gels with the sodium bicarbonate concentration increasing from 4 to 7% (*w/w*), respectively; B.—gel base; * CC_50_ values, calculated by extrapolation; N.D.—not determined.

## Data Availability

Data are contained within the article and supplementary materials.

## Supplementary Materials

The following supporting information can be downloaded at: https://www.mdpi.com/article/10.3390/pharmaceutics16111436/s1, Figure S1: Comparison of adhesion capacity of *L. crispatus* strains to HeLa, Hec-1A and VK2 E6/E7 cell lines.
