# Supplementary material for: Development and Characterization of Sodium Bicarbonate-Based Gel for Cytolytic Vaginosis
Source: Pharmaceutics. 2024 Nov 11;16(11):1436. doi: 10.3390/pharmaceutics16111436 (PMC11597264; doi:10.3390/pharmaceutics16111436)
Supplement: Supplementary file 1 [file pharmaceutics-16-01436-s001.zip › pharmaceutics-3269397-supplementary.pdf]

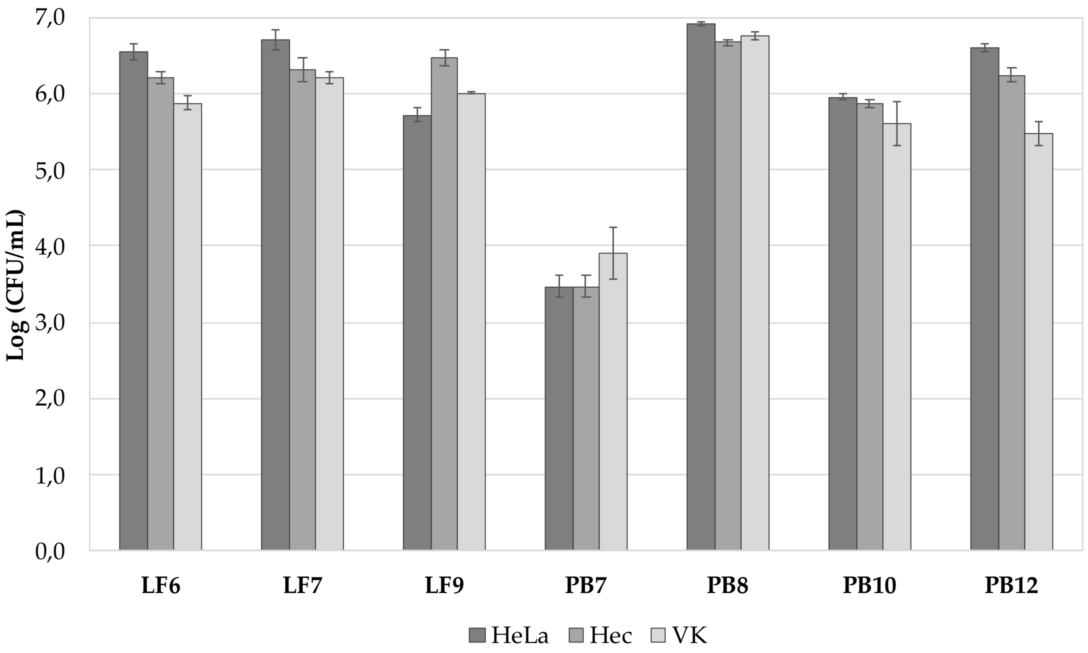

Figure S1. Comparison of adhesion capacity of *L. crispatus* strains to HeLa, Hec-1A and VK2 E6/E7 cell lines. *L. crispatus* strain PB7 has the lowest adhesion capacity to the three lines under study. On the other hand, *L. crispatus* strain PB8 is the strain with the highest adhesion capacity. In general, all strains preferentially adhere to the HeLa cell line.
